# Brain Regions Responsible for Tinnitus Distress and Loudness: A Resting-State fMRI Study

**DOI:** 10.1371/journal.pone.0067778

**Published:** 2013-06-25

**Authors:** Takashi Ueyama, Tomohiro Donishi, Satoshi Ukai, Yorihiko Ikeda, Muneki Hotomi, Noboru Yamanaka, Kazuhiro Shinosaki, Masaki Terada, Yoshiki Kaneoke

**Affiliations:** 1 Department of Anatomy and Cell Biology, Graduate School of Wakayama Medical University, Wakayama, Japan; 2 Department of System Neurophysiology, Graduate School of Wakayama Medical University, Wakayama, Japan; 3 Department of Neuropsychiatry, Graduate School of Wakayama Medical University, Wakayama, Japan; 4 Department of Otolaryngology-Head and Neck Surgery, Graduate School of Wakayama Medical University, Wakayama, Japan; 5 Wakayama-Minami Radiology Clinic, Wakayama, Japan; Yale University, United States of America

## Abstract

Subjective tinnitus is characterized by the perception of phantom sound without an external auditory stimulus. We hypothesized that abnormal functionally connected regions in the central nervous system might underlie the pathophysiology of chronic subjective tinnitus. Statistical significance of functional connectivity (FC) strength is affected by the regional autocorrelation coefficient (AC). In this study, we used resting-state functional MRI (fMRI) and measured regional mean FC strength (mean cross-correlation coefficient between a region and all other regions without taking into account the effect of AC (*rGC*) and with taking into account the effect of AC (*rGCa*) to elucidate brain regions related to tinnitus symptoms such as distress, depression and loudness. Consistent with previous studies, tinnitus loudness was not related to tinnitus-related distress and depressive state. Although both *rGC* and *rGCa* revealed similar brain regions where the values showed a statistically significant relationship with tinnitus-related symptoms, the regions for *rGCa* were more localized and more clearly delineated the regions related specifically to each symptom. The *rGCa* values in the bilateral rectus gyri were positively correlated and those in the bilateral anterior and middle cingulate gyri were negatively correlated with distress and depressive state. The *rGCa* values in the bilateral thalamus, the bilateral hippocampus, and the left caudate were positively correlated and those in the left medial superior frontal gyrus and the left posterior cingulate gyrus were negatively correlated with tinnitus loudness. These results suggest that distinct brain regions are responsible for tinnitus symptoms. The regions for distress and depressive state are known to be related to depression, while the regions for tinnitus loudness are known to be related to the default mode network and integration of multi-sensory information.

## Introduction

Subjective tinnitus is a common and disturbing event, characterized by the perception of phantom sound or noise in the ear or head without an external auditory stimulus [1]. Thus, in general, tinnitus is an entirely subjective experience that can only be described by patient reports. The prevalence of tinnitus is 10–15% of Western [2] and Japanese adults [3]. About 20% of patients require medical or psychiatric treatment since tinnitus frequently triggers psychological problems and reduces quality of life through depression [4], insomnia [5], and distress [6]. Symptoms of tinnitus [7] are evaluated by (1) otologic examination, (2) diagnostic pure tone audiometry for the assessment of hearing loss, (3) psychophysical measurements of tinnitus such as loudness match, pitch match, maskability and residual inhibition, and (4) validated questionnaires for the assessment of tinnitus-related distress such as THI (tinnitus handicap inventory) [8]. However, at present, there is no sufficient and established objective diagnostic test to evaluate the severity and other characteristics of tinnitus.

Involvement of the central nervous system (CNS) in the pathophysiology of tinnitus has been indicated by advances in neuroimaging techniques [9]–[12], such as positron emission tomography (PET), electroencephalography (EEG), magnetoencephalography (MEG), and functional magnetic resonance imaging (fMRI). These studies demonstrated the involvement of the primary auditory cortex and non-auditory brain areas such the anterior cingulate gyrus, anterior insula, amygdala, hippocampus, and parahippocampal region.

The CNS processes information using complex networks consisting of numerous brain regions. Various brain networks relevant to specific functions and diseases have been identified [13]–[20]. Electrophysiological studies have presented evidence of a modified CNS network in tinnitus subjects [21], [22]. While providing high temporal resolution, the MEG or EEG signal source is difficult to localize. It has been shown that correlation of low frequency fluctuations (0.01–0.1 Hz) of blood oxygenation level-dependent (BOLD) activity measured in fMRI study reflects brain network status (functional connectivity, FC) [13] [23]. Indeed, these fluctuations are shown to be coherent across widely separated (although functionally related) brain regions, constituting resting state networks [24]. Recently, Maudoux et al. and Burton et al. reported that FC between the auditory cortex and other cortices was altered in subjects with tinnitus [25], [26].

The previous studies estimated FC between selected seed regions such as the auditory cortex and other brain regions, resulting in probable oversight of additional underlying networks. Regional global connectivity (*rGC*) estimated by calculating each voxel’s mean FC (mean cross-correlation coefficients between a region and all other regions) allows a whole-brain approach to studying the pathophysiology of brain disorders. Similar concepts have been proposed by Scheinost et al. as the intrinsic connectivity distribution [27] and by Cole et al. as the global brain connectivity or weighted degree centrality [28], in which they computed the average connectivity of a region with the rest of the brain, to examine each region’s full range of connectivity. Although the *rGC* value of a region does not identify the specific networks in which the region is involved, *rGC* represents to what extent a given region coordinates with other brain regions. According to these recent theories and observations, we hypothesized that abnormal functionally connected regions in the CNS might underlie the pathophysiology in chronic subjective tinnitus. Therefore, resting-state fMRI and *rGC* analysis were applied to identify specific and crucial regions relevant to clinical parameters in subjects with tinnitus.

Cross-correlation coefficient is commonly used to evaluate the strength of FC between two regions. Because time series data is not random, the effective sample size of independent measurements across time must be estimated in order to calculate the statistical significance of FC [29]. The effective sample size can be estimated using the autocorrelation coefficient (AC) in the two regions as discussed in our previous study [20]. AC has physiological relevance [20], with low autocorrelation values distributed around the caudal brain regions and high values observed in the default mode network (DMN) regions, defined as “ a specific, anatomically defined brain system preferentially active when individuals are not focused on the external environment” [15]. In this study, we evaluated two types of regional global connectivity without sample size adjustment (*rGC*) or regional global connectivity with sample size adjustment (*rGCa*) in subjects with tinnitus.

## Materials and Methods

### Subjects

Twenty-four subjects suffering from mild to severe tinnitus who consulted the out-patient clinic of the Department of Otolaryngology-Head and Neck Surgery in Wakayama Medical University Hospital between August 2011 and April 2012 were re-enrolled in this study after providing written informed consent. This study was approved by the Wakayama Medical University Ethics Committee (No 962) and was performed according to the declarations of Helsinki. Subjects with a history of seizures, a suspected diagnosis of organic brain damage, brain tumor and psychiatric diseases were excluded. No subjects had a history of major depressive disorder (MDD) prior to the onset of tinnitus. The profiles of subjects (17 males and 7 females) are listed in Table 1. The mean age was 50.3 years (SD = 14.6 years, range 23–72 years) and the mean tinnitus duration was 50.8 months (SD = 102.9 months, range 3–400 months). Five subjects were prescribed anti-depressants (selective serotonin reuptake inhibitor: SSRI) or anti-anxiety drugs (benzodiazepines: BZD).

**Table 1:** Clinical profiles of subjects with tinnitus.

ID	Gender	Age(Y)	Duration (M)	THI	HAM-D	Medication	Hearing loss (dB)	Maximum loudness (dB HL)	Pitch (Hz)			Loudness (dB HL)		
				(0–100)	(0–52)				R	L	NL	R	L	NL
1	M	38	3	44	8	None	normal	25	12000	8000		20	25	
2	M	58	400	62	3	None	normal	50	10000	10000		40	50	
3	F	42	60	100	20	SSRI, BZD	normal	50		4000			50	
4	F	47	6	4	0	None	normal	40		1000			40	
5	M	58	24	16	2	None	30	40	4000	8000		40	25	
6	M	52	48	46	10	BZD	40	65	10000	10000		65	60	
7	M	72	36	66	2	None	60	65	125			65		
8	F	32	6	94	20	None	normal	35	250	250		30	35	
9	M	48	24	90	6	BZD	50	70	10000	8000		70	50	
10	M	67	24	74	15	BZD	50	80	250			80		
11	M	25	8	84	7	None	40	60	4000	4000		60	60	
12	M	69	360	58	1	None	70	70	10000	2000		70	60	
13	M	32	6	40	7	None	normal	35		12000			35	
14	F	63	6	36	0	None	50	50			4000			50
15	M	52	9	56	0	None	normal	40	12000	12000		40	30	
16	M	46	24	96	11	BZD	normal	50	12000	12000		25	50	
17	M	49	60	28	0	None	normal	60		10000			60	
18	M	60	12	30	1	None	40	55			12000			55
19	F	23	4	88	15	None	normal	35	12000	250		35	20	
20	M	58	12	80	12	None	60	60			3000			60
21	F	70	12	88	8	None	normal	45		125			45	
22	M	61	24	52	0	None	50	60		12000			60	
23	M	28	24	84	8	None	normal	35			12000			35
24	F	58	22	30	0	None	normal	35	8000	4000		15	35	

### Audiological Examination

Normal middle ear status was demonstrated by tympanometry and otoscopy. All subjects underwent audiological testing to determine hearing levels (AA-78, RION, Tokyo, Japan). Pure tones ranging from 250 Hz to 12 kHz were presented to each ear until the threshold of detection was reached. Thirteen subjects showed normal hearing and 11 subjects had mild to moderate hearing loss. The subjects underwent additional audiological testing to identify the best match to the perceived frequency of their tinnitus. The tinnitus pitch was determined by presenting pure tones with increasing frequency from low to high to patients until they formed a match, then from high to low, and then averaging the two matches. Although many other methods are used to measure subjectively perceived loudness of tinnitus, the maximum intensity of the tinnitus (Loudness) was determined as the loudness balance value (hearing level, dB HL; sound volume equivalent to that which the subject feels), but not the sensation level (dB SL; subtracting the hearing threshold value from the loudness balance value) as discussed later.

The laterality of tinnitus was found to be left (6), right (2), bilateral (12) and no laterality (The subject said “I feel that my tinnitus originates around the center of the brain”) (4). (Table 1).

### Assessment of Severity and Tinnitus-related Distress

The severity of tinnitus and related distress were measured using the established tinnitus handicap inventory (THI) originally developed by Newman [8]. The subjects were also interviewed using the Structured Clinical Interview for DSM-IV and the 17-item Hamilton Depression Rating Scale (HAM-D) [30] by a psychiatrist.

### Image Acquisition

Image acquisition and data processing were described previously [20]. Briefly, a 3 Tesla MRI (PHILIPS, The Netherlands) using a 32-channel head coil (SENSE-Head-32CH) was used to obtain each subject’s brain structural and resting state functional images. The mechanical sounds produced during the MRI procedure were masked by use of both earpieces and headphones. The following parameters were used for T1-weighed anatomical images: TR = 7 ms, TE = 3.3 ms, FOV = 220 mm, Matrix scan = 256, slice thickness = 0.9 mm, and flip angle = 10°. The following parameters were used for functional data using a gradient-echo echo-planar pulse sequence sensitive to BOLD contrast [31]: TR = 3000 ms, TE = 30 ms, FOV = 192 mm, Matrix scan = 64, slice thickness = 3 mm, and flip angle = 80°. Three runs, each with 105 volumes (for 5 min 15 s), were administered to each subject. During acquisition, the subjects were asked to stay awake with their eyes closed.

### MRI Data Analysis

SPM8 (http://www.fil.ion.ucl.ac.uk/spm) and in-house software developed with MATLAB (Mathworks, Natick, MA, USA) were used to preprocess fMRI data. The first 3 volumes of each fMRI acquisition run were discarded in order to eliminate equilibration effects, leaving 102 consecutive volumes per session. To remove gross head motion, rigid body translation and rotation were performed, and spatial normalization was achieved by 12-parameter affine transformation to the International Consortium for Brain Mapping Echo-Planar Imaging template in SPM8. Sessions with large motion (>2°) were excluded. Each image was resampled to 2-mm isotropic voxels and spatially smoothed using an 8-mm full width at half maximum Gaussian kernel. Structural images were also normalized and resampled to extract time series data for the cerebrospinal fluid (CSF), white matter (WM), and gray matter (GM), which were used to reduce non-physiological noise in BOLD signals (see below). Each subject’s three tissue images (CSF, WM and GM) were generated using SPM8 with a probability threshold of 90%.

CompCor [32] and global signal regression [33] were used to exclude the signals unrelated to brain function, such as brain tissue fluctuations due to head motion, cardiac activity, and respiration. Briefly, CompCor includes the following steps: 1) identification of voxels showing the highest temporal variation (top 2%), 2) principal component analysis (PCA) of these voxels and voxels within CSF and WM, 3) identification of the PCA components accounting for a significant proportion of the variance in the data, and 4) exclusion of the identified signal time course for each voxel using linear regression. Temporal filtering (band-pass, ranging from 0.01 to 0.1 Hz) removed constant offsets and linear trends over each run. The 102 preprocessed images obtained from each session were converted into single four-dimensional (time and three spatial data) images, and then the data from 3 sessions were used for the following analysis.

The *rGC* map was created by calculating each voxel’s weighted degree. Weighted degree indicates the mean cross-correlation function at the same time (lag 0) between a seed voxel’s signal time course and all other voxels’ signal time courses [16], [18]. *rGC* represents the mean FC (thus, weighted degree) between a voxel and all the other voxels in each GM voxel. For computational efficiency, voxels within the GM were down sampled to 6 mm isotropic voxels before the calculation of the FC. *rGC* at voxel *i* (6×6×6 mm) was defined as:
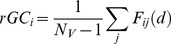
(1)where *N_V_* is number of GM voxels, *d* is the slice-scan-time lag (ranging from 0 to 3000 ms) depending on the image slice number difference between voxel *i* and *j*, and *F_ij_* is normalized cross-correlation function between voxel *i* and *j* in the GM:
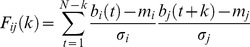
(2)

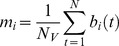
(3)

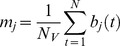
(4)

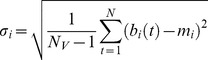
(5)

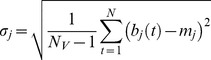
(6)where *b_i_* and *b_j_* are BOLD signals at voxels *i* and *j*, respectively.

*rGCa* with sample size adjustment using autocorrelation was calculated as follows. First, the autocorrelation function for each voxel of the functional GM volumes was calculated. Then, the first order (lag 1) AC (*r*) was calculated for each voxel using the following equations:
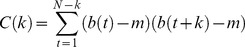
(7)

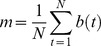
(8)

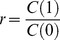
(9)where *C(k)* is autocorrelation at lag *k* of *N* sample data (*N* = 102, in this study). Note that C(0) is equal to the signal’s variance, and that dividing C(1) by C(0) gives a proper correlation (*r*) between −1 and 1.

Second, the effective sample size (*N′*) between two voxels was determined:
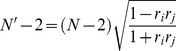
(10)where *r_i_* and *r_j_* are the respective first order AC of the two time series at voxels *i* and *j*.

Third, the normalized cross-correlation function was determined as done for *rGC*. Each *F_ij_* value was transformed to *t* value using the effective sample size:
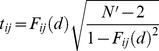
(11)


Finally, *rGCa* at voxel *i* (6×6×6 mm) was calculated:
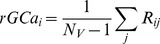
(12)where *R_ij_* is the *Z* transform of the *t_ij_* with the effective sample size [29].

The three dimensional presentation of *rGC* and *rGCa* was created using MRIcron [34], which was also used to estimate the anatomical localization of the regions of interest.

Connectivity-clinical parameter analysis was performed as follows. To remove the effects of a subject’s age, hearing level (normal or impaired) and medication (not medicated or medicated), we computed the partial correlations between “THI” (the scores of tinnitus-related distress), “HAM-D” (Hamilton Depression Rating Scale), or “Loudness” (loudness balance value in each subject) and *rGC* or *rGCa* across subjects using Spearman’s method (considering the data are not normally distributed) in a voxel-wise manner. Then, to evaluate the significance of the *rGC* or *rGCa*-Loudness, -THI, or -HAM-D correlation in each voxel, the statistic *t* was determined as,
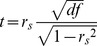
(13)where *r_s_* denotes Spearman’s correlation coefficient by ranks between *rGC* or *rGCa* and the “Loudness” or “THI”, and *df* is the degree of freedom. Here, *df* was equal to 22.

To correct for multiple comparisons, we set the single voxel threshold at *p*<0.01 (∣t(22) 2.51>∣) and used a minimum cluster size of 2808 mm^3^ (13 adjacent voxels), which provided a corrected threshold of *p*<0.05 as determined by Monte Carlo simulation (AlphaSim by D. Ward in AFNI software. Parameters were as follows: single *p* value = 0.01; FWHM = 8 mm; Cluster connection radius: rmm = 12.00; with a mask of the respective composite FC map).

## Results

### Assessment of Clinical Parameters in Tinnitus

The distribution of THI ranged from 4 to 100 (mean = 60.3, SD = 27.8), while that of HAM-D was from 0 to 20 (mean = 6.5, SD = 6.4), and that of Loudness was from 25 to 80 dB HL (mean = 50.4 dB HL, SD = 14.2 dB HL) (See Table 1).

**Figure 1:**
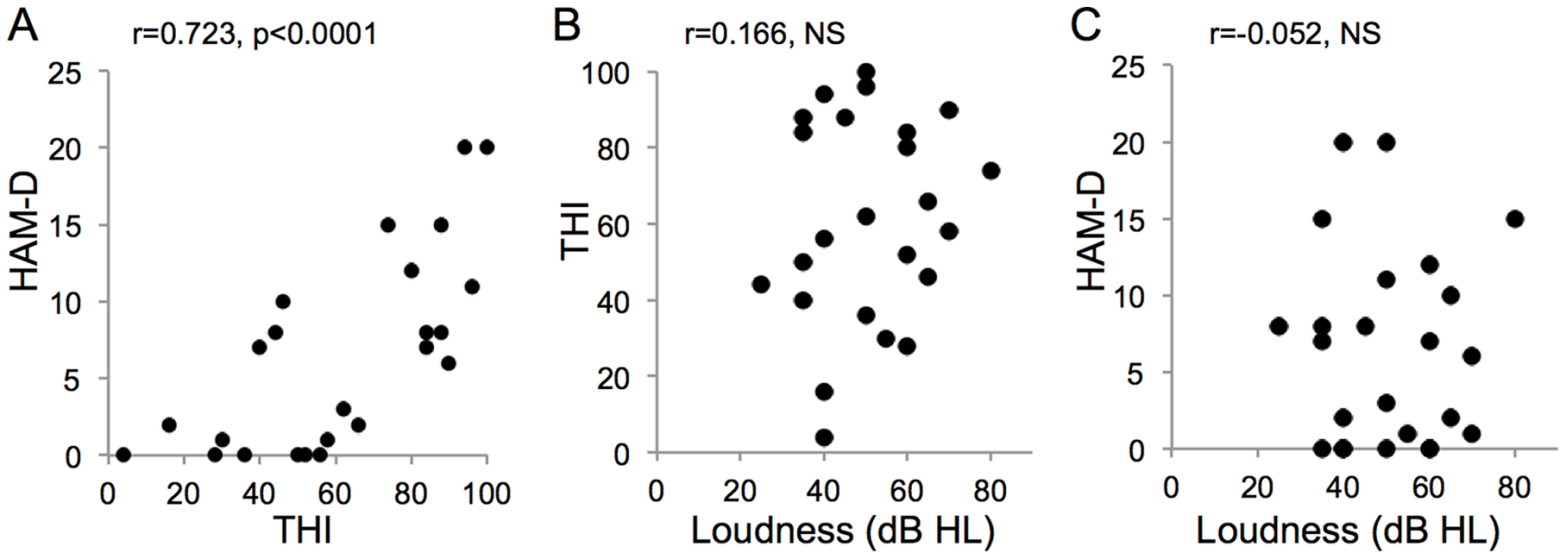
Correlation between THI, HAM-D, and Loudness. (A) Correlation between the scores of tinnitus-related distress (THI) and Hamilton Depression Rating Scale (HAM-D). Significant positive correlation was observed (*r* = 0.723, *p*<0.0001). (B)Correlation between the maximum intensity of the tinnitus, “Loudness” determined as the loudness balance value (dB HL) and THI. They were not correlated significantly (*r* = 0.166, NS). (C) Correlation between the maximum intensity of tinnitus, “Loudness” determined as the loudness balance value (dB HL) and HAM-D. They were not correlated significantly (*r* = −0.052, NS).

As shown in Figure 1A, the scores of THI and HAM-D were correlated significantly (*r* = 0.723, *p*<0.0001). However, the scores of THI or HAM-D and Loudness were not correlated significantly (Figures 1B and 1C).

### Brain Regions Responsible for HAM-D

**Figure 2:**
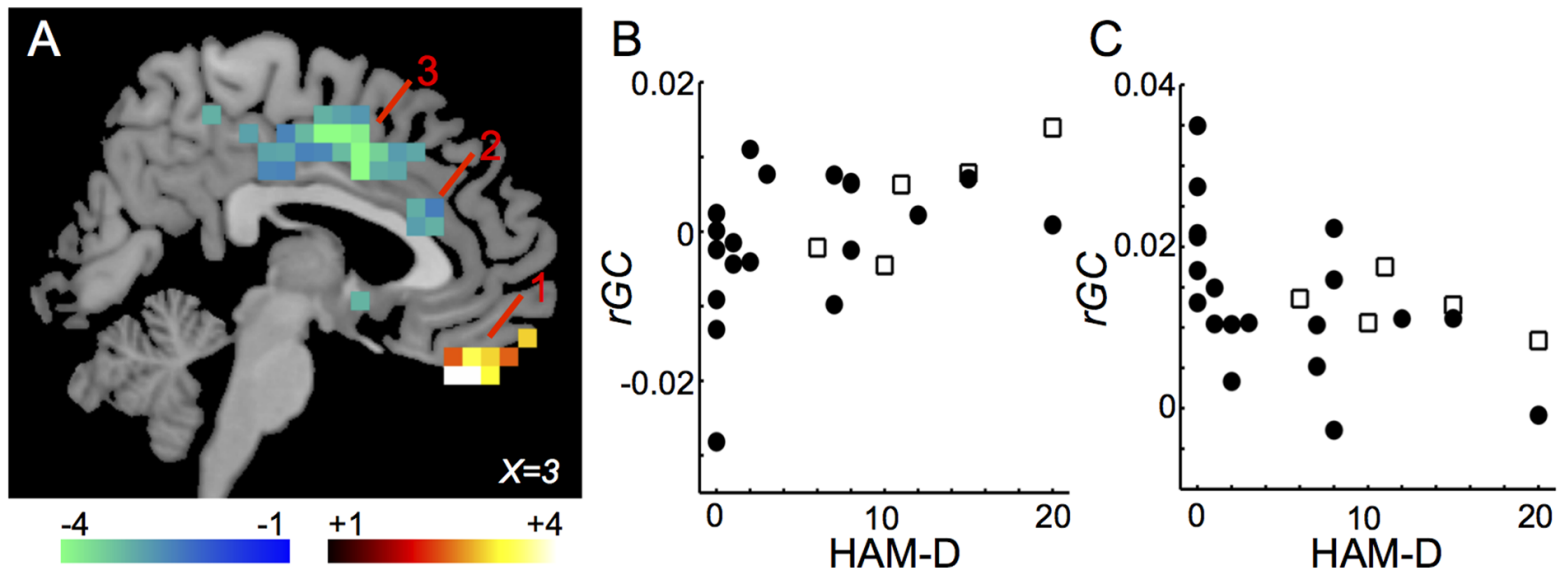
Regions in which HAM-D was correlated significantly with *rGC*. (A) Only voxels (6x6x6 mm) with significant t-values (*p*<0.05 corrected for multiple comparisons) are shown. 1: the right rectus gyrus (BA11, 25); 2: the right anterior cingulate gyrus (BA24, 32); 3: the right middle cingulate gyrus (BA23, 24). (B) Distribution of HAM-D-*rGC* plots in the left rectus gyrus between subjects with and without medications (*r* = 0.516, *p* = 0.00415). Closed circle indicates the subject without medication and opened square indicates the subject with medication. (C) Distribution of HAM-D-*rGC* plots in the right middle cingulate gyrus between subjects with and without medications (*r* = −0.472, *p* = 0.00856). Closed circle indicates the subject without medication and opened square indicates the subject with medication.

When the sample size was not adjusted, *rGC* in the bilateral rectus gyri (BA11, 25) (Figure 2A, “1”) were correlated positively with HAM-D. *rGC* in the bilateral anterior cingulate gyri (BA24, 32) (Figure 2A, “2”) and the bilateral middle cingulate gyri (BA23, 24) (Figure 2A, “3”) were correlated negatively with HAM-D. Distributions of HAM-D-*rGC* plots in the left rectus gyrus and in the right middle cingulate gyrus between subjects with and without medications are shown in Figures 2B and 2C. The *rGC*-HAM-D correlations are listed in Table 2. The MNI coordinates in the tables refer to the peak of correlation.

**Table 2:** The *rGC*-HAM-D correlations.

Region	MNI			BA	*r*	*p*
	x	y	z			
rectus gyrus	−6	42	−24	11	0.516	0.00415
anterior cingulate gyrus	1	25	22	24	−0.478	0.00788
middle cingulate gyrus	3	4	38	24	−0.472	0.00856

**Figure 3:**
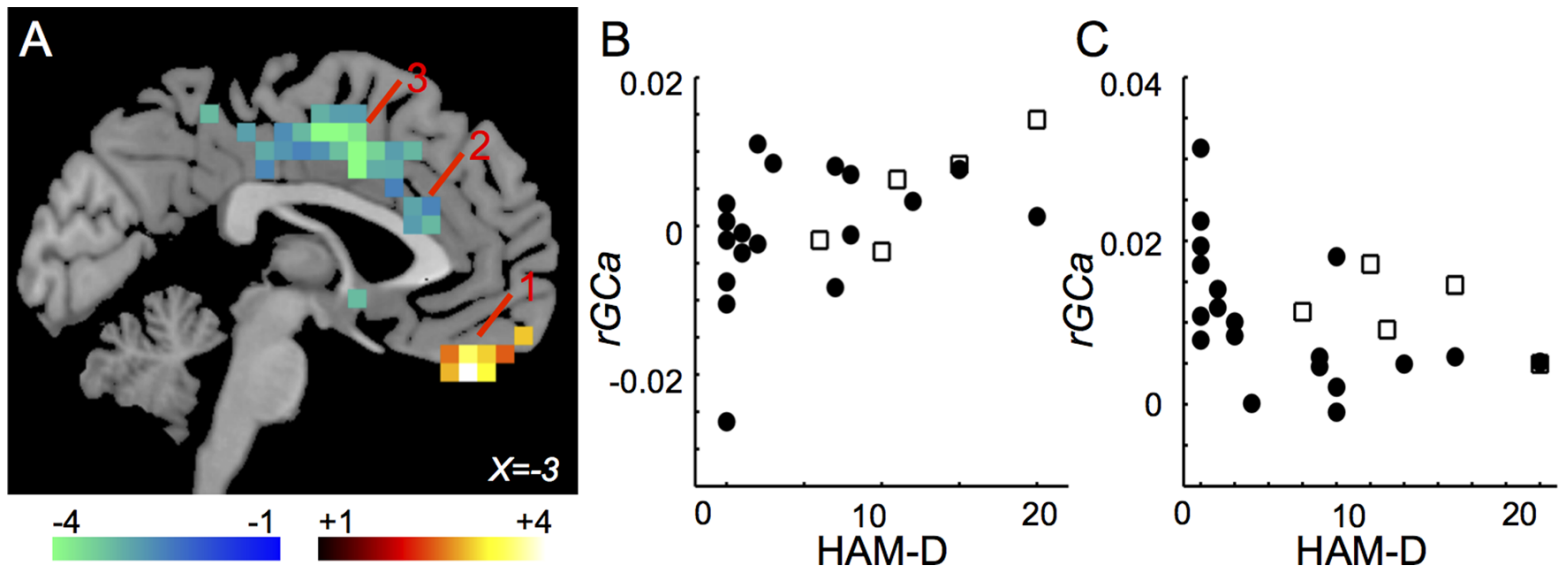
Regions in which HAM-D was correlated significantly with *rGCa*. (A) Only voxels (6x6x6 mm) with significant t-values (*p*<0.05 corrected for multiple comparisons) are shown. 1: the left rectus gyrus (BA11, 25); 2: the left anterior cingulate gyrus (BA24, 32); 3: the left middle cingulate gyrus (BA23, 24). (B) Distribution of HAM-D-*rGCa* plots in the left rectus gyrus between subjects with and without medications (*r* = 0.537, *p* = 0.00280). Closed circle indicates the subject without medication and opened square indicates the subject with medication. (C) Distribution of HAM-D-*rGCa* plots in the left middle cingulate gyrus between subjects with and without medications (*r* = −0.478, *p* = 0.00780). Closed circle indicates the subject without medication and opened square indicates the subject with medication.

*rGCa*, which are calculated using effective sample size, in the same brain regions as *rGC* were correlated with HAM-D. *rGCa* in the bilateral rectus gyri (BA11, 25) (Figure 3A, “1”) were correlated positively with HAM-D, while those in the bilateral anterior cingulate gyri (BA24, 32) (Figure 3A, “2”) and the bilateral middle cingulate gyri (BA23, 24) (Figure 3A, “3”) were correlated negatively with HAM-D. Distributions of HAM-D-*rGCa* plots in the left rectus gyrus and in the left middle cingulate gyrus between subjects with and without medications are shown in Figures 3B and 3C. The *rGCa*-HAM-D correlations are listed in Table 3.

**Table 3:** The *rGCa*-HAM-D correlations.

Region	MNI			BA	*r*	*p*
	x	y	z			
rectus gyrus	−1	46	−24	11	0.537	0.0028
anterior cingulate gyrus	−1	27	18	24	−0.471	0.0088
middle cingulate gyrus	−1	8	34	24	−0.478	0.0078

### Brain Regions Responsible for THI

**Figure 4:**
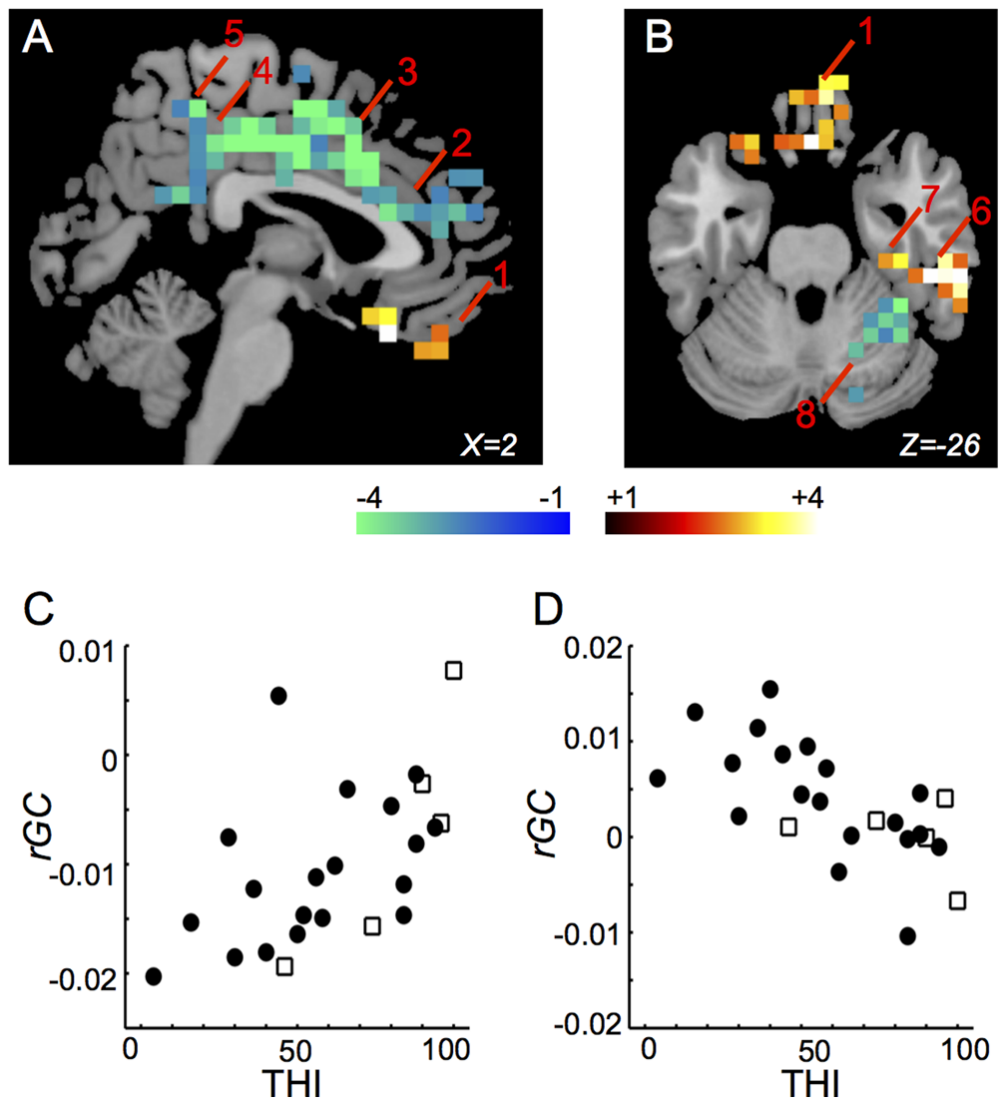
Regions in which THI was correlated significantly with *rGC*. (A) and (B) Only voxels (6x6x6 mm) with significant t-values (*p*<0.05 corrected for multiple comparisons) are shown. 1: the right rectus gyrus (BA11, 25); 2: the right anterior cingulate gyrus (BA24, 32); 3: the right middle cingulate gyrus (BA23, 24); 4: the right posterior cingulate gyrus (BA23, 26); 5: the left precuneus; 6: the right inferior temporal gyrus (BA20); 7: the right fusiform gyrus (BA20); 8: the right cerebellar hemisphere (BA37). (C) Distribution of THI-*rGC* plots in the right inferior temporal gyrus between subjects with and without medications (*r* = 0.629, *p* = 0.000383). Closed circle indicates the subject without medication and opened square indicates the subject with medication. (D) Distribution of THI-*rGC* plots in the right cerebellar hemisphere between subjects with and without medications (*r* = −0.690, *p* = 6.8E-05). Closed circle indicates the subject without medication and opened square indicates the subject with medication.

*rGC* in the bilateral rectus gyri (BA11, 25) (Figures 4A and 4B, “1”), the right inferior temporal gyrus (BA20) (Figure 4B, “6”), and the right fusiform gyrus (BA20) (Figure 4B, “7”) were correlated positively with THI. *rGC* in the bilateral anterior cingulate gyri (BA24, 32) (Figure 4A, “2”), the bilateral middle cingulate gyri (BA23, 24) (Figure 4A, “3”), the bilateral posterior cingulate gyri (BA23, 26) (Figure 4A, “4”), the bilateral precuneus (Figure 4A, “5”), the right inferior parietal gyrus (BA40), the right middle temporal gyrus (BA20, 21), the bilateral occipital gyri (BA19), and the right cerebellar hemisphere (BA37) (Figure 4B, “8”) were correlated negatively with THI. Distributions of THI-*rGC* plots in the right inferior temporal gyrus and in the right cerebellar hemisphere between subjects with and without medications are shown in Figures 4C and 4D. The *rGC*-THI correlations are listed in Table 4.

**Table 4:** The *rGC*-THI correlations.

Region	MNI			BA	*r*	*p*
	x	y	z			
rectus gyrus	3	25	−24	11	0.518	0.004
anterior cingulate gyrus	3	27	18	24	−0.546	0.0024
middle cingulate gyrus	1	−13	39	23	−0.569	0.00149
posterior cingulate gyrus	1	−40	32	23	−0.438	0.0144
precuneus	−2	−42	40		−0.422	0.0177
right inferior parietal gyrus	37	−44	52	40	−0.506	0.0049
right middle temporal gyrus	61	−25	−7	21	−0.512	0.00448
right inferior temporal gyrus	57	−30	−26	20	0.629	0.000383
right fusiform gyrus	37	−25	−26	20	0.545	0.00236
inferior occipital gyrus	−49	−69	−13	19	−0.624	0.000432
right cerebellar hemisphere	37	−45	−26	37	−0.69	0.000068

**Figure 5:**
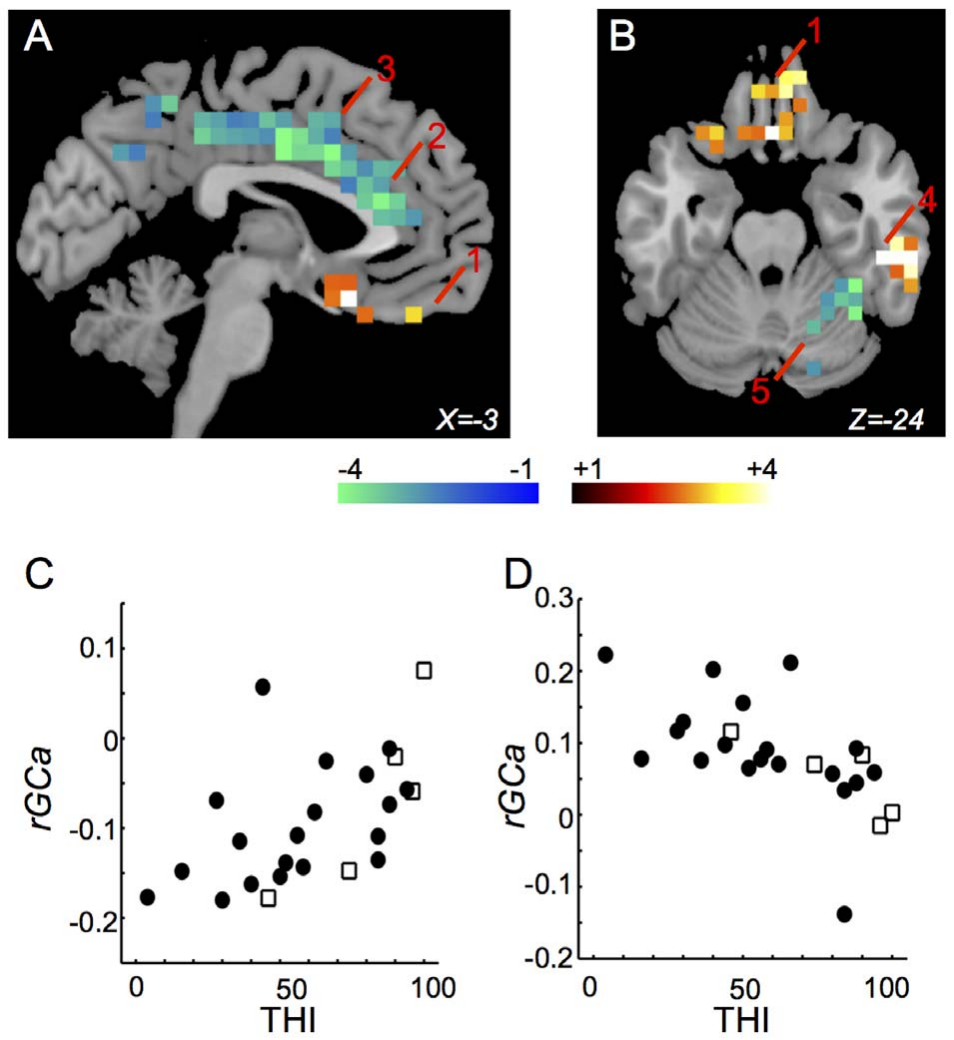
Regions in which THI was correlated significantly with *rGCa*. (A) and (B) Only voxels (6x6x6 mm) with significant t-values (*p*<0.05 corrected for multiple comparisons) are shown. 1: the left rectus gyrus (BA11, 25); 2: the left anterior cingulate gyrus (BA24, 32); 3: the left middle cingulate gyrus (BA23, 24); 4: the right inferior temporal gyrus (BA20); 5: the right cerebellar hemisphere (BA37). (C) Distribution of THI-*rGCa* plots in the right inferior temporal gyrus between subjects with and without medications (*r* = 0.634, *p* = 0.000335). Closed circle indicates the subject without medication and opened square indicates the subject with medication. (D) Distribution of THI-*rGCa* plots in the right cerebellar hemisphere between subjects with and without medications (*r* = −0.684, *p* = 8.21E-05). Closed circle indicates the subject without medication and opened square indicates the subject with medication.

Some of the brain regions found for *rGC* showed a significant relationship between *rGCa* and THI. *rGCa* in the bilateral rectus gyri (BA11, 25) (Figures 5A and 5B, “1”), and the right inferior temporal gyrus (BA20) (Figure 5B, “4”) were correlated positively with THI. *rGCa* in the bilateral anterior cingulate gyri (BA24, 32) (Figure 5A, “2”), the middle cingulate gyri (BA23, 24) (Figure 5A, “3”), the right inferior parietal gyrus (BA40), the right postcentral gyrus (BA40), and the right cerebellar hemisphere (BA37) (Figure 5B, “5”) were correlated negatively with THI. Distributions of THI-*rGCa* plots in the right inferior temporal gyrus and in the right cerebellar hemisphere between subjects with and without medications are shown in Figures 5C and 5D. The *rGCa*-THI correlations are listed in Table 5.

**Table 5:** The *rGCa*-THI correlations.

Region	MNI			BA	*r*	*p*
	x	y	z			
rectus gyrus	−3	18	−18	11	0.471	0.00873
anterior cingulate gyrus	−3	30	16	24	−0.546	0.00238
middle cingulate gyrus	−3	12	34	24	−0.528	0.00336
right inferior parietal gyrus	31	−49	48	40	−0.575	0.00131
right postcentral gyrus	32	−35	48	40	−0.569	0.0015
right inferior temporal gyrus	61	−31	−24	20	0.634	0.000335
right cerebellar hemisphere	31	−48	−24	37	−0.684	0.0000821

### Brain Regions Responsible for Loudness

**Figure 6:**
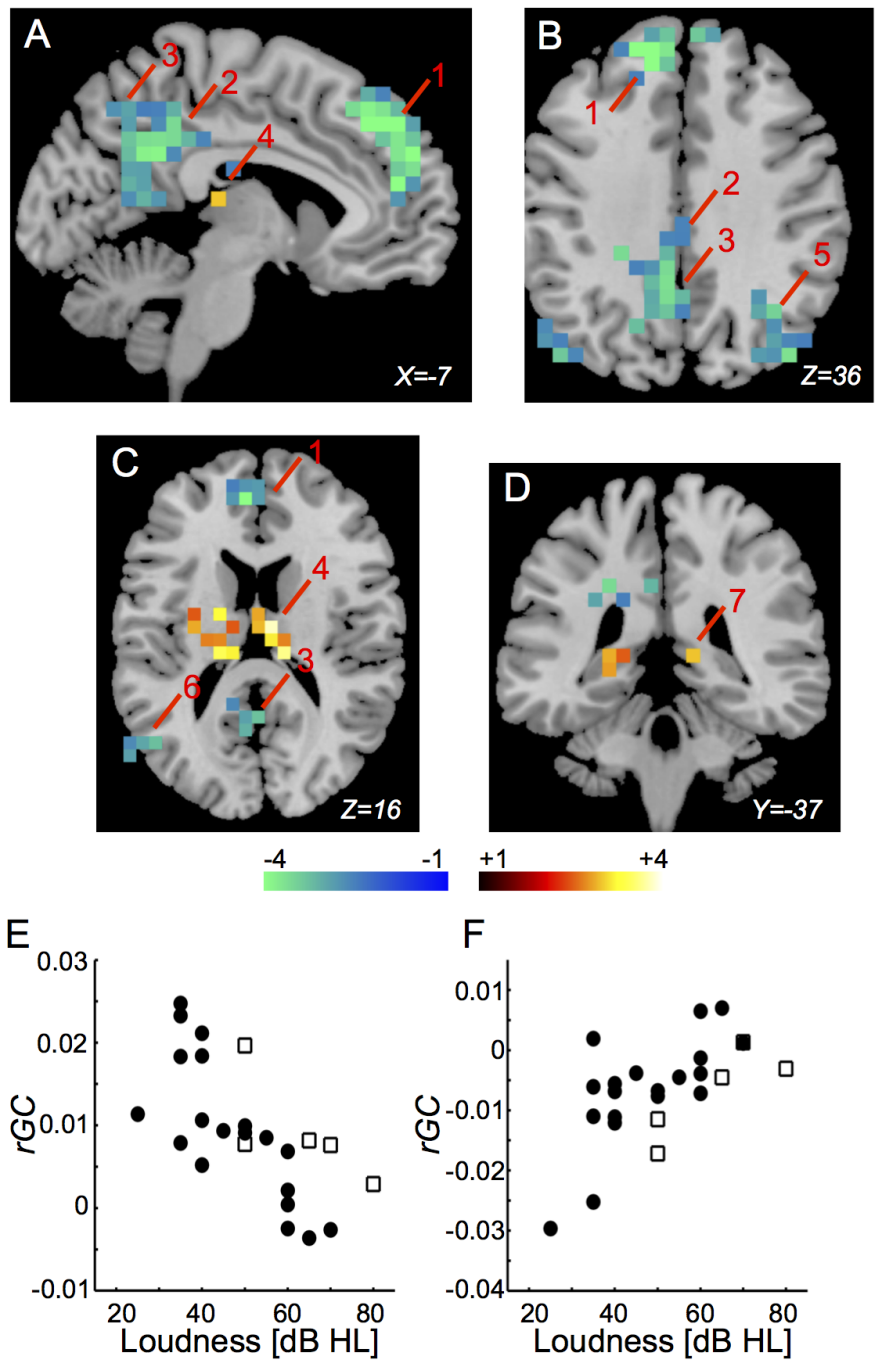
Regions in which Loudness was correlated significantly with *rGC*. (A)–(D) Only voxels (6x6x6 mm) with significant t-values (*p*<0.05 corrected for multiple comparisons) are shown. 1: the left medial superior frontal gyrus (BA9, 10, 32); 2: the left posterior cingulate gyrus (BA23, 26); 3: the left precuneus; 4: the left thalamus; 5: the right medial occipital gyrus (BA19); 6: the left middle temporal gyrus (BA39); 7: hippocampus. (E) Distribution of Loudness-*rGC* plots in the left medial superior frontal gyrus between subjects with and without medications (*r* = −0.748, *p* = 8.6E-06). Closed circle indicates the subject without medication and opened square indicates the subject with medication. (F) Distribution of THI-*rGC* plots in the right thalamus between subjects with and without medications (*r* = 0.603, *p* = 0.000704). Closed circle indicates the subject without medication and opened square indicates the subject with medication.

*rGC* in the bilateral medial superior frontal gyri (BA9, 10, 32) (Figures 6A–6C, “1”), the bilateral posterior cingulate gyri (BA23, 24) (Figures 6A, 6B, “2”), the left precuneus (Figures 6B and 6C, “3”), the left middle temporal gyrus (BA39) (Figure 6C, “6”), the bilateral angular gyri (BA19, 39), and the right middle occipital gyrus (BA19) (Figure 6B, “5”) were correlated negatively with Loudness. *rGC* in the bilateral thalamus (Figures 6A, 6C, “4”) and the bilateral hippocampus (BA27) (Figure 6D, “7”) were correlated positively with Loudness. Distributions of Loudness-*rGC* plots in the left medial superior frontal gyrus and in the right thalamus between subjects with and without medications are shown in Figures 6E and 6F. The *rGC*-Loudness correlations are listed in Table 6.

**Table 6:** The *rGC*-Loudness correlations.

Region	MNI			BA	*r*	*p*
	x	y	z			
medial superior frontal gyrus	−7	48	41	9	−0.748	0.0000086
posterior cingulate gyrus	−5	−49	30	23	−0.533	0.00306
left precuneus	−8	−53	38		−0.548	0.0023
left middle temporal gyrus	−49	−69	24	39	−0.506	0.00497
angular gyrus	45	−76	37	39	−0.564	0.00165
right middle occipital gyrus	39	−75	36	19	−0.574	0.0014
thalamus	11	−23	11		0.603	0.000704
hippocampus	−21	−38	6	27	0.467	0.00931

**Figure 7:**
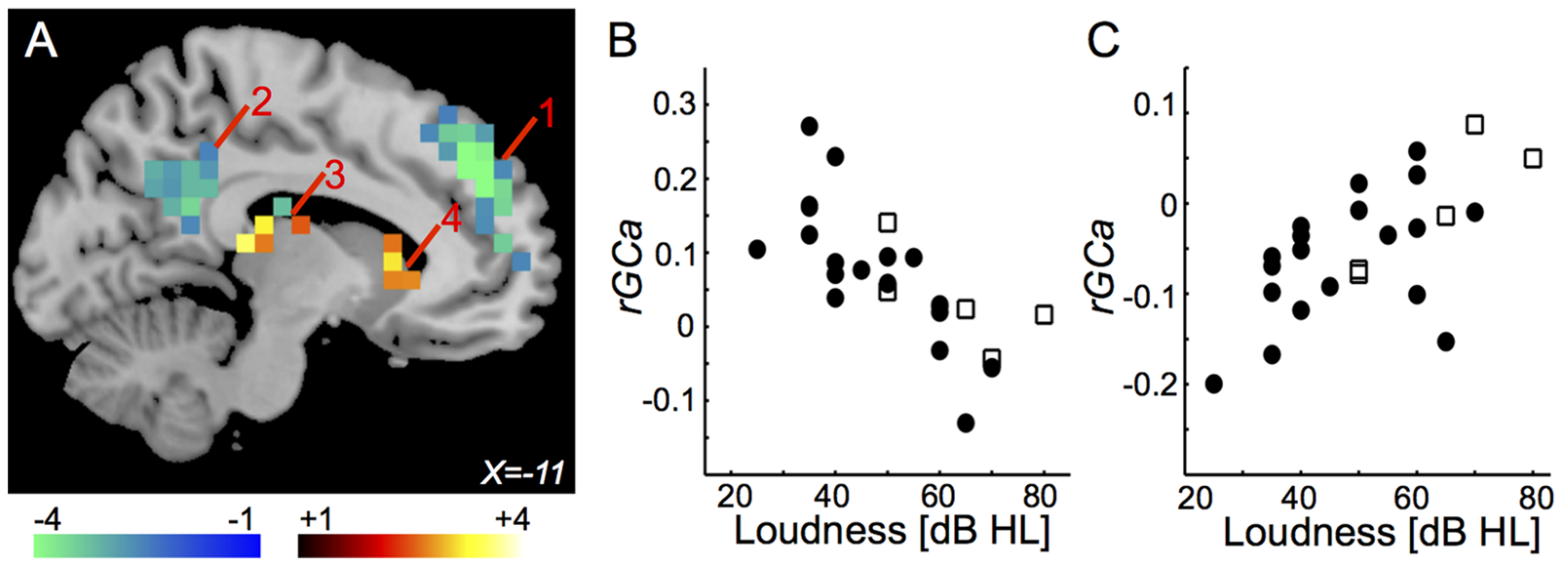
Regions in which Loudness was significantly correlated with *rGCa*. (A) Only voxels (6x6x6 mm) with significant t-values (*p*<0.05 corrected for multiple comparisons) are shown. 1: the left medial superior frontal gyrus (BA9, 10, 32); 2: the left posterior cingulate gyrus (BA23, 26); 3: the left thalamus; 4: the left caudate. (B) Distribution of Loudness-*rGCa* plots in the left medial superior frontal gyrus between subjects with and without medications (*r* = −0.855, *p* = 2.58E-08). Closed circle indicates the subject without medication and opened square indicates the subject with medication. (C) Distribution of Loudness-*rGCa* plots in the left thalamus between subjects with and without medications (*r* = 0.591, *p* = 0.00094). Closed circle indicates the subject without medication and opened square indicates the subject with medication.

Some of the brain regions found for *rGC* showed a significant relationship between *rGCa* and Loudness. *rGCa* in the left medial superior frontal gyrus (BA9, 10, 32) (Figure 7, “1”) and the left posterior cingulate gyrus (BA23, 24) (Figure 7, “2”) were correlated negatively with Loudness. *rGCa* in the bilateral thalamus (Figure 7, “3”), the bilateral hippocampus (BA27), and the left caudate (Figure 7, “4”) were correlated positively with Loudness. Distributions of Loudness-*rGCa* plots in the left medial superior frontal gyrus and in the left thalamus between subjects with and without medications are shown in Figures 7B and 7C. The *rGCa*-Loudness correlations are listed in Table 7.

**Table 7:** The *rGCa*-Loudness correlations.

Region	MNI			BA	*r*	*p*
	x	y	z			
left medial superior frontal gyrus	−11	46	34	9	−0.855	2.58E-08
left posterior cingulate gyrus	−11	−48	22	23	−0.567	0.00156
thalamus	−17	−29	6		0.591	0.00094
hippocampus	−20	−36	6	27	0.471	0.00873
left caudate	−17	18	6		0.465	0.00963

## Discussion

### Assessment of Clinical Parameters in Tinnitus

Tinnitus subjects differ regarding the intensity and laterality of their phantom sounds, hearing levels, duration of the symptoms, and the magnitude of psychological impacts. In this study, we evaluated clinical profiles by tinnitus-related distress, depression, and tinnitus loudness, respectively (See Material and Methods). A strong positive correlation was demonstrated between the scores of THI and HAM-D. As none of the subjects had episodes of MDD prior to the onset of tinnitus, tinnitus was considered to be the primary cause of depressive state in these subjects. We also reconfirmed that the scores of distress and Loudness were not correlated significantly, consistent with numerous other reports [6], [35], [36].

Reliable and appropriate evaluation of tinnitus loudness is difficult. Although there are many modified methods to evaluate tinnitus loudness, all of them have some limitations [37]–[41]. Subjectively perceived loudness of tinnitus has been recorded by numeric rating scales that typically range from 0 or 1 (low loudness) to 10 (high loudness). It is difficult to compare the values among subjects. For example, it is not known whether one subject’s rate 8 indicates the tinnitus sound intensity equivalent to another subject’s rate 8. We used the maximum loudness balance value (maximum sound volume equivalent to that which the patient feels in audiological testing) as the “Loudness” of the patients, and distributions of Loudness were from 25 to 80 dB HL. If the sensation level (SL; subtraction of the hearing threshold value from the loudness balance value) was used as the “Loudness”, tinnitus loudness might be underestimated because of recruitment phenomenon [37]–[40]. In most cases, the levels of tinnitus intensity were under 20 dB SL, which were much less than that of their subjective feelings. In some cases, the intensity of tinnitus was estimated to be below 5 dB SL. Although Loudness as estimated by hearing levels might be overestimated, it is closer to that of subjective feelings rather than that by sensation level. We estimated the connectivity-clinical parameter analysis using Loudness estimated by sensation level. The detected regions (data not shown) were scattered in the brain with poor correlations, and the physiological implications of these regions could not be explained. When Loudness as estimated by hearing levels was adopted, strong correlations were observed in the localized regions with physiological significances as discussed below. These points support the validity of Loudness used in this study.

### General Remarks on the Results in rGC Analysis

Many of the regions identified by *rGC* analysis are known to play a role in auditory and emotional processing as discussed below. These regions are similar, in part, to regions identified in tinnitus by other modalities such as alteration of BOLD fMRI signals, regional blood flow estimated by SPECT, and neuronal activities estimated by PET, EEG, and MEG in response to stimulation or in comparison between tinnitus subjects and controls, as discussed later. Most studies indicate involvement of the primary auditory cortex in subjects with tinnitus. PET and fMRI studies have reported elevated blood flow and BOLD signals both in steady-state metabolism and sound-evoked responses [42]. In this study, the primary auditory cortex (Heschl gyrus) was not identified as a region in which *rCG* or *rGCa* were significantly correlated with THI, HAM-D and Loudness. This apparent discrepancy may be due to differences in the analytical approach. Most previous studies investigated the difference between tinnitus patients and non-tinnitus controls, while our study compared the differences among the tinnitus subjects. If alterations of FC in the primary auditory cortex were equivalent among the tinnitus subjects, no relationship between *rGC/rGCa* and tinnitus-related symptoms would be detected.

If AC in a given voxel is large, *rGC* is over-estimated as described in the introduction. Thus, intrinsic regional activity may be the neuronal basis of the regions that showed significant relationship with tinnitus-related symptoms only in *rGC*. In contrast, *rGCa* reflects the net cross-correlation coefficients between that region and all other regions. The numbers of the regions in association with *rGCa* were smaller than those in *rGC* (Tables 4 and 5, Tables 6 and 7). Therefore, we consider that *rGCa* is more likely to strictly reflect CNS network change in relation to tinnitus. Thus, we discuss the physiological and pathological implication of the regions associated with *rGCa.*

### Brain Regions Associated with THI and HAM-D

A strong positive correlation between THI and HAM-D suggests that tinnitus subjects with high THI scores suffer as severely as subjects with MDD (Figure 1A). Significant positive correlations between *rGCa* and THI or HAM-D were observed in the bilateral rectus gyri (BA11, 25), while significant negative correlations were observed in the bilateral anterior and bilateral middle cingulate gyri (BA23, 24, 32) (Figure 3 and 5, Table 3 and 5). These regions overlap, in part, with the regions associated with MDD.

Structural, functional imaging and therapeutic studies in MDD [43]–[45] indicate abnormalities in the frontal lobes including regions of the dorsolateral and ventrolateral prefrontal cortex (especially BA9, BA46 and BA47), as well as orbital frontal cortices (especially BA10, BA11, BA32) and the anterior part of cingulate gyrus (BA24 and BA25). MDD showed a reduction of GM volumes in the bilateral anterior cingulate gyri, the rectus gyri, and the orbitofrontal cortices, as well as the basal ganglion, thalamus, and hippocampus [43], [44]. In response to treatment for depression, decreased blood flow was observed in the subcallosal cingulate gyrus (SCC)(BA25), the ventral-most segment of the cingulate gyrus, as well as the orbital and medial frontal cortices (BA10, 11), and increased blood flow in the dorsolateral prefrontal cortex (BA9/46), dorsal anterior cingulate gyrus (BA24), and posterior cingulate gyrus (BA31) [45]. It was demonstrated in tinnitus subjects that the volume of GM was reduced and the volume of WM was increased in the ventromedial prefrontal cortex compared with normal controls [46]. Therefore, the ventromedial prefrontal cortex is considered a key region involved in tinnitus.

Our studies also demonstrated the involvement of the cingulate gyrus, a pivotal part of the limbic system, in tinnitus-related distress and depression, as demonstrated in other studies as well. In previous studies, tinnitus subjects showed a stronger activation of BOLD fMRI signals to tinnitus-related sentences in the anterior, middle, and posterior cingulate gyri, retrosplenial cortex, and insula when compared with healthy controls [47]. Resting-state EEG studies demonstrated that in subjects with serious tinnitus distress, more synchronized alpha activity was observed in the anterior cingulate gyrus, the insula, parahippocampal area, and amygdala, and less alpha synchronized activity was found in the posterior cingulate gyrus, precuneus, and dorsal lateral prefrontal cortex [48]. PET studies indicated that tinnitus distress was correlated positively with activation of the bilateral posterior inferior temporal gyri and bilateral posterior parahippocampal areas [49]. These regions overlap, in part, with the regions in which significant THI-*rGCa* correlations were observed, although the relationship with *rGCa* and regional neuronal activity is not known.

### Brain Regions Responsible for Loudness

The regions that are relevant to Loudness were as follows. Significant negative correlations between *rGCa* and Loudness were observed in the left medial superior frontal gyrus (BA9, 10, 32) and the left posterior cingulate gyrus (BA23, 26), while significant positive correlations were observed in the bilateral thalamus, the bilateral hippocampus (BA27), and the left caudate (Figure 7 and Table 7).

The thalamus is the main relay center between the cerebral cortex and various peripheral sensory systems. The primary auditory thalamic inputs to the auditory cortex originate in the medial geniculate complex, but other nuclei such as the suprageniculate, posterior, peripeduncular, and pulvinar nuclei are also involved in the auditory thalamocortical projection [50]. The positive correlation between Loudness and *rGC*a in the bilateral thalamus suggests possible pathological implications in the perception of phantom sound. Interestingly, direct electrical stimulation in the ventral intermediate nucleus of the thalamus ameliorated the loudness symptoms of tinnitus in some subjects [51]. The caudate nucleus, a striatal center for sensorimotor integration, is also the potential target of direct electrical stimulation for the treatment of tinnitus. It is reported that direct electrical stimulation in the caudate nucleus modified tinnitus loudness [52], [53].

The posterior cingulate gyrus (BA23, 26) and medial superior frontal gyrus (BA 9, 10, 32) are known as parts of the DMN. Negative correlations between *rGCa* and Loudness were observed in the left posterior cingulate gyrus (*r* = −0.567, *p* = 0.00156) and the left medial superior frontal gyrus (*r* = −0.855, *p* = 2.58E-8), suggesting that the degree of Loudness may affect function of the DMN. The function of the DMN may be disturbed depending on the degree of Loudness, or the DMN may be affected in subjects with tinnitus by some inputs outside the DMN (possibly from tinnitus generating regions).

It was reported that the volume of GM decreased in the superior and medial frontal gyri in subjects with hearing loss compared to normal hearing controls, although the involvement of tinnitus was not clear [54]. These regions also showed differential responses to pure tone stimulation between bilateral hearing loss with tinnitus and bilateral hearing loss without tinnitus [55]. Another report showed that the left medial superior frontal gyrus was activated by laser stimulation of the tympanic membrane [56]. These observations implicate that the left medial superior frontal gyrus is involved in normal or abnormal auditory function. Although conclusive interpretations are difficult to establish with the current information, these anatomical and functional data suggest that the left medial superior frontal gyrus is a critical region that integrates multi-sensory information including auditory sensation and the pathophysiology in tinnitus perception.

### The Other Brain Regions Responsible for Tinnitus

*rGCa* analysis suggested the involvement of other critical brain regions such as the parietal association areas (BA 40), the temporal cortex (BA 20), hippocampus, (BA27) and cerebellum (BA37). The right inferior parietal gyrus (BA40), the right postcentral gyrus (BA40), the right inferior temporal gyrus (BA20), and the right cerebellar hemisphere (BA37) were involved in tinnitus distress, while the hippocampus (BA27) was involved in tinnitus loudness. Several lines of studies indicated involvement of these regions in the pathophysiology of tinnitus. Decreased regional cerebral blood flow in the right frontal lobe (BA 45), the left parietal lobe (BA 39), and the left visual association cortex (BA 18) was observed in tinnitus subjects compared with non-tinnitus subjects [57]. Significant reduction of GM was demonstrated in tinnitus subjects in the right inferior colliculus and in the left hippocampus [58]. The cerebellum is also involved in the auditory system. Activation in response to sound was observed in the lateral cerebellum and the primary auditory cortex in PET study [59]. In particular, an increase in cortical synaptic activation with 40-Hz stimulation was observed in the posterolateral portion of both cerebellar hemispheres, lateral to the paravermian region, in Crus II [60]. The same group, using fMRI, reported that input from the auditory superior temporal gyrus and superior temporal sulcus to the cerebellum was enhanced selectively at gamma-band frequencies around 40Hz [61].

### Limitations

Our subject group was heterogenous, with wide distributions of age and duration of tinnitus. In addition, cases with both moderate hearing loss and normal hearing were included. Minor tranquilizers or anti-depressants were prescribed in five cases. These drugs can affect the cerebral FC. Indeed, BZD have been reported to affect the cerebral FC [62], [63]. We compared the distribution of clinical parameter*-rGC* or *-rGCa* plots between subjects with and without medications as shown in Figures 2–7. The distribution trends seemed to be similar between subjects with and without medications. To mitigate these confounding variables, we controlled for the effects of subject age, hearing loss and medication by computing the partial correlations using Spearmen’s method between THI, HAM-D, or Loudness and *rGC* or *rGCa* across subjects in a voxel-wise manner.

In order to exclude the signals unrelated to brain function (such as brain tissue fluctuations due to head motion, cardiac activity, and respiration), we used CompCor [32] and global signal regression [33], and excluded the sessions with large head motion. It is possible that the residual noise could affect the FC analysis as recent studies [64]–[67] have shown. However, the effect of head motion appears unlikely to contribute to the correlation between *rGC/rGCa* and clinical parameters at the *localized* regions observed in this study because head motion would influence regional connectivities in the whole brain.

Evaluation of tinnitus loudness is difficult especially where hearing loss has occurred as discussed above. By use of the loudness balance value without subtraction from the hearing threshold, significant correlations between *rGCa* and Loudness were observed in several regions, some of which were in accordance with previous knowledge of the auditory system and tinnitus, as discussed above.

The distress of most tinnitus subjects is generally mild to moderate (scores less than 56 on the THI scale). In a Japanese community-based investigation, severe tinnitus annoyance was observed in 2.9% (man) and 3.2% (women) among tinnitus patients [3]. In contrast, 9 cases (38%) were rated at catastrophic level (scores more than 78 on the THI) and 4 cases (17%) were rated at severe level (58–76 on the THI) in this study. It is possible that significant correlations between THI or HAM-D and *rGCa* in the anterior, middle cingulate gyri, and rectus gyri were detected because of sample deviations in severity.

Another problem is the association of hyperacusis, or diminished sound level tolerance. Gu et al demonstrated that subjects with hyperacusis showed elevated activation in the auditory midbrain, thalamus, and the primary auditory cortex compared with subjects with normal tolerance [68]. Hwang reported that idiopathic hyperacusis exhibited sound-elicited activation in the frontal lobes (superior, middle, interior frontal gyri) and occipital lobes (precuneus, cuneus, superior occipital gyrus, lingual gyrus or fusiform gyrus) [69]. In addition, the laterality of subjective tinnitus was varied. In this study, the maximum loudness balance value of the subject was used for calculating the correlation with *rGC* and *rGCa* independent of laterality. This may influence the detection of the locus with significant *rGC* and *rGCa*, especially in its laterality and autocorrelation. Further study including sample sizes sufficient for classifying subjects based on the hearing levels, laterality, age and other confounding variables will be required.

Although MRI noise levels were reduced as far as possible, ambient noise may be a limiting factor in auditory studies using MRI techniques. The goal of this study was to detect alterations of the CNS network related to tinnitus distress, depression, and tinnitus loudness. Only subjects with tinnitus were investigated without a control healthy group. Accordingly, caution is required in concluding that FC is related to tinnitus per se. Furthermore, it remains to be elucidated by interventional studies whether the observed correlations reflect causal relations or pure epiphenomena.

### Conclusions

We reconfirmed that tinnitus distress or depression and tinnitus loudness are independent clinical factors. In accordance with these clinical observations, different regions were involved in tinnitus distress, depression and tinnitus loudness, respectively. MDD related regions, such as the bilateral rectus gyri, the bilateral anterior, and middle cingulate gyri were associated with THI and HAM-D. In contrast, Loudness showed a relationship with *rGCa* values in the regions related to the DMN and integration of multi-sensory information such as the left medial superior frontal gyrus, the left posterior cingulate gyrus, the bilateral thalamus, the bilateral hippocampus, and the left caudate. Further studies are required to elucidate how these regions are involved in tinnitus symptoms.
